# Regulation of iron ion homeostasis in *Staphylococcus aureus* and its impact on physiology and virulence

**DOI:** 10.1128/mbio.01110-26

**Published:** 2026-06-09

**Authors:** Gustavo Rios-Delgado, Jeffrey M. Boyd

**Affiliations:** 1 Department of Biochemistry and Microbiology, Rutgers, the State University of New Jersey 242612, New Brunswick, New Jersey, USA; The Ohio State University, Columbus, Ohio, USA

**Keywords:** iron, Fur, IsrR, Fpa, metalloprotein, metalloenzyme, metalloregulation, pathogenesis, metabolism, virulence regulation, *Staphylococcus aureus*, metals

## Abstract

Bacteria use transcription factors and small regulatory RNAs as modulators of transcription and translation to respond effectively to diverse environmental and internal stressors. Nearly all bacteria require iron (Fe), and in host niches, pathogenic bacteria encounter a shortage of available Fe ions; their response to Fe depletion significantly affects their physiology and pathogenesis. Here, we review how the bacterial pathogen *Staphylococcus aureus* uses the ferric uptake regulator (Fur), Fur protein antagonist (Fpa), AcnA, and iron-sparing response regulator (IsrR) to regulate gene expression in response to changes in cytosolic Fe levels. Fur is a transcription factor that uses Fpa and Fe(II) ions to regulate the transcription of genes, including those involved in Fe uptake. Upon Fe limitation,Fpa mediates alleviation of Fur repression. This enables the expression of the IsrR, a non-coding RNA. IsrR binds to and represses translation of mRNAs encoding proteins that require Fe or are involved in Fe-dependent processes, thereby mediating an Fe-sparing response. In response to Fe depletion, Fur and IsrR promote increased Fe uptake and a general shift toward fermentative metabolism for energy conservation. IsrR also directly mediates the translational repression of detoxification mechanisms against oxidative stress and directly or indirectly alters the expression of virulence factors. Fe depletion also impacts the iron-sulfur enzyme aconitase by enriching for the apo-form, which moonlights as an RNA-binding protein (RBP) with regulatory activity. We showcase how transcription factors, non-coding RNAs, and RBPs control bacterial Fe homeostasis, highlighting their roles as critical mediators of bacterial stress responses and virulence.

## NUTRITIONAL IMMUNITY AND *STAPHYLOCOCCUS AUREUS* IRON ACQUISITION STRATEGIES

The bacterium *Staphylococcus aureus* is a common opportunistic human pathogen and a significant public health concern worldwide (1). *S. aureus* causes an array of clinically presenting infections, including potentially fatal bacteremia and pneumonia (2). This review summarizes recent findings on how *S. aureus* responds to iron (Fe) deprivation, a common and significant stress encountered in the host. Iron is an essential nutrient for bacterial pathogens like *S. aureus*, and hosts go to great lengths to prevent bacteria from accessing it during infection. Bacteria often respond to Fe restriction by altering the expression of genes used in Fe acquisition and virulence. We postulate that understanding the physiological changes that *S. aureus* undergoes during Fe limitation is necessary to comprehend pathogenesis.

Iron is a growth-limiting nutrient for nearly all pathogens, including *S. aureus*. Iron is used as a cofactor for multiple enzymes involved in diverse cellular processes, including central metabolism, DNA synthesis, and repair (3). Bacterial reliance on Fe-requiring proteins is exploited by vertebrate hosts during infections, which limit the availability of metal ions, setting up a battle between the bacterium trying to access Fe and the host trying to prevent access (reviewed in reference 4). The process by which hosts actively limit the availability of metal ions is known as nutritional immunity (5). Most of the human Fe pool is complexed in hemoglobin-bound heme inside erythrocytes (6). In serum, host proteins haptoglobin and hemopexin bind and protect hemoglobin and heme, respectively, while Fe ions are bound by transferrin and lactoferrin (7–11). The excess non-chelated Fe in cells is bound by ferritin for protection and storage (12). Within phagocytic compartments of innate immune cells, Fe is exported via efflux pumps, thereby reducing pathogen access (13, 14). Additionally, during infection, the host increases plasma transferrin and decreases dietary Fe absorption, further restricting Fe availability (15).

To overcome host-mediated Fe sequestration, *S. aureus* reprograms its gene expression to produce a range of Fe-acquisition gene products (16). The main *S. aureus* Fe-uptake strategies include a heme uptake system (Isd), and Fe-binding metallophores. It also increases expression of cytotoxins to lyse leukocytes (e.g., LukED) or erythrocytes (e.g., Hla), presumably increasing access to the pools of protein-associated Fe and heme Fe found in hemoglobin (17). The released heme, hemoglobin, and haptoglobin-bound hemoglobin are recognized by cell wall-anchored surface receptors (IsdB, IsdA, IsdC, and IsdH) from the Isd heme acquisition system, which are architecturally positioned to interact with extracellular heme (reviewed in reference 18). Heme is ultimately transferred through the Isd receptors to the lipoprotein IsdE. The heme is then passed from IsdE to the IsdF permease complex for internalization (19, 20). The IsdEF transporter is an ATP-binding cassette (ABC) transporter, comprised of a substrate-binding protein (IsdE), a membrane-spanning permease (IsdF), and an associated cytosolic ATP hydrolase (FhuC) (21). On the inner leaflet of the cytosolic membrane, the monooxygenases IsdI and IsdG liberate the Fe(II) from the heme. *S. aureus* mutants lacking *isdA, isdB, isdC, isdG,* and *isdI* have reduced bacterial loads in kidneys or hearts in a systemic model of infection, suggesting a role for heme uptake in pathogenesis (22, 23). Aside from their role in heme uptake, Isd proteins also promote resistance to host innate immune defenses (24, 25). *S. aureus* also codes for the Fep system, which is proposed to acquire Fe from heme (26, 27).

During heme scarcity, Fe can be scavenged as Fe(III) from host Fe-binding proteins, such as transferrin and lactoferrin, using Fe-chelating siderophores. Siderophores are excreted low-molecular-weight compounds with a high affinity for Fe(III) (28). *S. aureus* synthesizes two carboxylate-type siderophores: staphyloferrin A and staphyloferrin B (29, 30). Once bound to Fe, staphyloferrin A and B are imported through the ABC transporters Hts and Sir, respectively (31). Aside from its own siderophores, *S. aureus* can import exogenously produced siderophores through the Fhu and Sst ABC transport systems (32, 33). *S. aureus* also contains two siderophore-independent transporters (Feo and Fep) hypothesized to import Fe(II) (26, 34). Lastly, Fe ions can be scavenged by staphylopine, a broad-spectrum metallophore capable of binding Cu, Ni, Co, Zn, or Fe with differing affinities (35–37). Although staphylopine has a greater affinity for Zn(II) and Mn(II) than Fe ions, the system is repressed by both the Zn(II)-responsive transcriptional regulator Zur and the Fe(II)-responsive transcriptional regulator Fur, demonstrating that both Fe(II) and Zn(II) depletion increases staphylopine expression (35). The regulatory control of staphylopine by Fur supports a role for staphylopine in Fe uptake or a connection between Zn/Mn ion homeostasis and Fe ion homeostasis.

## TRANSCRIPTIONAL REGULATION BY THE FERRIC UPTAKE REGULATOR (Fur)

To coordinate the expression of Fe uptake genes while avoiding the consequences of Fe overload, many bacteria control Fe homeostasis using the ferric uptake transcriptional regulator (Fur) (17). Fur has been shown to regulate Fe homeostasis in numerous bacteria, but extensive biochemical work has only been conducted on Fur from gram-negative bacteria. The canonical model for gram-negative Fur function is primarily that of an Fe(II)-dependent repressor of genes encoding Fe uptake proteins during Fe-replete growth conditions. Several studies have also demonstrated that Fur can act as a transcriptional activator (38, 39).

Fur is a homodimer where each protomer contains an N-terminal DNA-binding domain and a C-terminal dimerization domain (40, 41). Fur has been shown to bind to various divalent metals *in vitro*, including Fe (40, 42), and Fe is proposed to be the physiological co-repressor, but this has not been definitively demonstrated (reviewed in reference 40). Each Fur monomer contains binding sites for two to three metal ions, including a regulatory metal-binding site believed to be associated with a labile Fe(II) (41). *Escherichia coli* Fur contains a structural zinc-binding site thought to aid in folding and dimerization (43). Studies with *Magnetospirillum gryphiswaldense* Fur showed that the metal-binding sites were essential for gene regulation *in vivo* (44).

In the recognized Fur-dependent regulation model, under Fe-replete conditions, Fur binds Fe(II) at its regulatory metal-binding site, inducing a conformational change in which the N-terminal DNA-binding domains move in a “caliper” manner to a closed conformation, thereby increasing affinity for specific DNA sequences (44). Fur recognizes and binds to a consensus DNA sequence known as a Fur box, which is usually overlapping with the −10 and −35 RNA polymerase recognition sequences upstream of the transcriptional start site (45). Therefore, Fur-DNA binding often prevents RNA polymerase from binding to or recognizing the operator, resulting in transcriptional repression. During growth in Fe-restricted conditions, it has been hypothesized that Fur loses its regulatory Fe(II) co-repressor, causing a structural change to an “open” conformation in which the affinity for DNA decreases, leading to Fur release from the DNA and allowing transcription of target genes.

It was recently demonstrated that the *E. coli* Fur protein can bind a 2Fe-2S iron-sulfur (Fe-S) cluster *in vitro* using two cysteine ligands per monomer, at the location previously described as a zinc-binding site (46). The *E. coli* Fur co-purified with an intact Fe-S cluster, and Fur purified from an *E. coli* strain lacking the SufA and IscA Fe-S cluster trafficking proteins had decreased Fe-S cluster occupancy, suggesting that Fe-S binding takes place *in vivo*. Fur purified from an *E. coli* strain lacking IscU, the scaffold for Fe-S cluster synthesis by the Isc system, lacked an Fe-S cluster. An *iscU* mutant exhibited a decreased ability to repress transcription from a Fur-regulated promoter *in vivo* as the Fe concentration in the growth medium increased (47). The overall impact of Fe-S cluster binding by Fur on Fur-dependent transcriptional regulation *in vivo* and whether Fur-Fe-S cluster binding is conserved among bacteria, including Isc-lacking gram-positive bacteria, remain unclear.

The sensing of Fe availability and control of Fe homeostasis by Fur are conserved across gram-positive and gram-negative bacteria. However, biochemical and physiological studies of Fur-dependent regulation have primarily been conducted in gram-negative bacteria; thus, little is known about the metal-binding properties of Fur in gram-positive bacteria. There is no evidence that Fe functions as a co-repressor or that regulatory metal sites exist. According to RegPrecise, Fur is predicted to bind around 20 gene or operon operators in *S. aureus*, directly controlling the expression of nearly 50 genes. These genes are primarily associated with Fe acquisition (48). Aside from transcriptionally repressing Fe uptake, *S. aureus* Fur also broadly impacts gene expression, including activating Fe-containing proteins through the small regulatory RNA (sRNA) iron-sparing response regulator (IsrR), as discussed below.

## THE FUR PROTEIN ANTAGONIST (Fpa)

Work in *Bacillus subtilis* found that *fpa* (*ylaN*) is essential for growth under standard laboratory conditions, and a strain with decreased *fpa* transcription exhibited phenotypes similar to those of strains with decreased transcription of *sufCDSUB*, which codes for the essential Fe-S cluster synthesis system (3, 49). A *B. subtilis fpa* deletion strain could be generated if the complex growth medium was supplemented with a high concentration of Fe ions, further demonstrating a link between Fe and *fpa* (49). Supplementing the growth medium with Fe was not required to create an *S. aureus fpa* mutant, and a *fpa* mutant did not have a noticeable growth defect unless Fe was limited (50). Suppressor analyses were conducted by plating the *S. aureus fpa* mutant on solid medium containing a non-growth-permissive concentration of the divalent metal chelator 2,2-dipyridyl. The second-site mutations that permitted growth mapped to *fur* and were null mutants, demonstrating that the low-Fe sensitivity phenotype of the *fpa* mutant could be suppressed by derepressing the Fur regulon. Further experimentation confirmed that the antagonistic activity of Fpa is required to express genes repressed by Fur, as Fe restriction is not enough to alleviate Fur repression in an *fpa* mutant (50). The overexpression of *fpa* led to Fur derepression of the Fur-repressed *dhbA* in *B. subtilis* (51). In *S. aureus*, overexpression of *fpa* led to accumulation of cell-associated Fe, likely by derepressing Fur-regulated Fe acquisition systems (50).

Fpa has not been identified in genomes outside of the Firmicutes. Phylogenetic analyses of Fur from organisms that do and do not possess Fpa demonstrated that, once acquired, Fpa is retained in genomes. These analyses also suggested that Fpa and Fur have been co-evolving, leading to the hypothesis that their functions are tethered (50). In support of this, cross-linking work in *B. subtilis* identified an *in vivo* Fpa-Fur interaction that included the Lys74 of Fur, which is near a proposed regulatory metal-binding site (52, 53). Likewise, when Fpa was immunoprecipitated from *B. subtilis* cells, Fur was the primary co-isolated protein (50). Bacterial two-hybrid analyses demonstrated that the *B. subtilis* Fpa and *B. subtilis* Fur interact in *E. coli,* and binding occurred when the growth medium was supplemented with either 0.1 µM or 500 µM Fe(III), suggesting that growth with excess Fe does not hinder Fpa-Fur interaction (51). Additional two-hybrid analyses in *S. aureus* confirmed that *S. aureus* Fpa and Fur proteins interact*,* and again, no differences were noted upon Fe starvation or growth with excess Fe (50). Biochemical work using recombinantly produced *S. aureus* Fur demonstrated that it bound to the DNA operator of the Fur-regulated gene *isdC*, and no difference in binding affinity was noted when additional Mn(II) or Fe(II) was added to the binding mixture. When Fpa was titrated into the Fur and DNA binding mixture, there was a decreased affinity of Fur for DNA as the concentration of Fpa was increased (50).

X-ray structural analyses suggest that *S. aureus* Fpa is a dimer (54), which was supported by bacterial two-hybrid analyses (50). *S. aureus* Fpa binds one Fe(II) per monomer with a *K*_d_ of approximately 2 µM (50). In *E. coli* cells, approximately 1% of the total intracellular Fe pool is predicted to be only loosely associated with proteins or metabolites (55). During aerobic growth, this labile Fe ion pool has been demonstrated to be approximately 20 µM (56). If *S. aureus* has a similarly sized labile Fe pool during Fe-replete growth, it is likely that Fpa is in the holo form.

The association of Fpa with Fe(II) alters the secondary structure and improves thermal stability, suggesting that Fe(II) stabilizes the protein. Normalized X-ray absorption near edge spectroscopy demonstrated that Fpa bound Fe(II) exclusively (50). Extended X-ray absorption fine structure analyses demonstrated that Fe(II) was bound using oxygen and/or nitrogen ligands, suggesting amino acid ligation (50).

**Fig 1:**
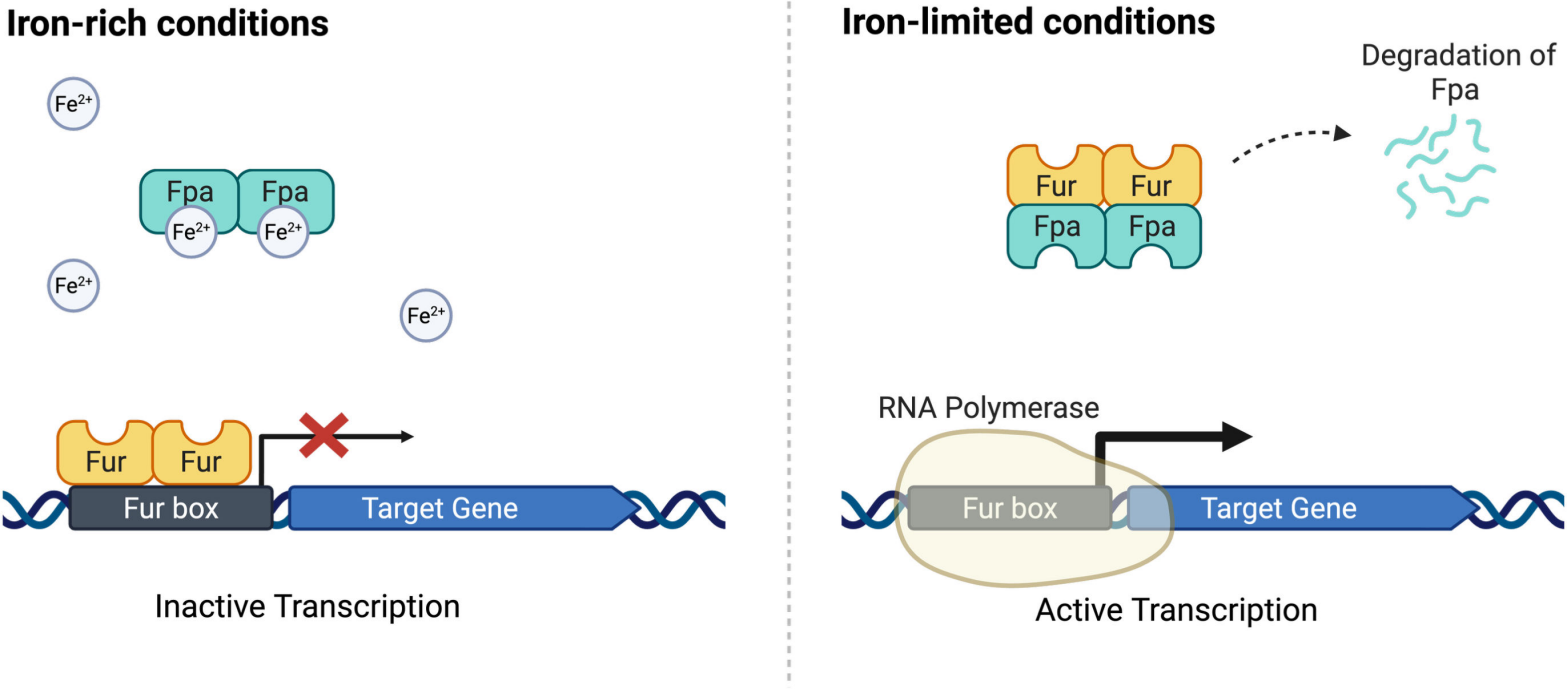
Model for gene regulation by Fur and Fpa. During growth in Fe-rich conditions, Fpa is metalated with Fe(II), and Fur binds to a consensus DNA sequence known as a Fur box. Fur boxes are typically located upstream of Fur-target genes in the operator regions, and Fur binding often prevents transcription by DNA-directed RNA polymerase. In Fe-limited conditions, Fpa forms a complex with Fur, decreasing the affinity of Fur for DNA. This alleviates Fur repression and increases transcription of Fur-target genes. In our model, Fur-bound Fpa is degraded, allowing the system to reset and maintain homeostasis. It is currently unknown whether *S. aureus* Fur associates with Fe(II) or what role Fe binding by Fpa plays. Created in BioRender (G. Rios, 2026, https://BioRender.com/k6qzbyd).

The findings above have led to a model of Fpa and Fur regulation of Fe homeostasis in Firmicutes (Fig. 1). During growth in Fe-replete conditions, Fpa is expressed and binds to Fe(II), leading to a decreased ability of Fpa to interact with Fur. During growth in Fe-deplete conditions, apo-Fpa forms a complex with Fur, preventing Fur from associating with gene operators and increasing transcription of the Fur regulon. Structural analysis using AlphaLink predicts that Fpa interferes with Fur dimerization and reorients the DNA-binding domains (51). The reorientation of these domains by Fpa could explain how Fpa antagonizes Fur and prevents Fur-DNA binding. Future work is necessary to determine how Fpa is regulated, how Fe impacts the antagonizing activity of Fpa, and how Fpa-Fur interaction leads to derepression of Fur target genes. Recent work in proteobacteria has also demonstrated that Fur regulation can involve protein-protein interactions that antagonize Fur-DNA binding (57). Examples of Fur antagonists include EIIA^Ntr^ in *Salmonella enterica* and the SlyD/YdiV pair in *Escherichia coli (58, 59*). It will be interesting to determine whether these proteins and Fpa have similar mechanisms for altering Fur-dependent regulation.

## INDIRECT IMPACT OF Fur ON GENE EXPRESSION

Although Fur transcriptional control is primarily associated with Fe acquisition loci in *S. aureus*, proteomic and transcriptomic analyses revealed that the absence of Fur or growth in Fe-limiting conditions impacts a variety of cellular processes, including central metabolism and the oxidative stress response, demonstrating that the impact of Fur-dependent regulation goes beyond Fe uptake (16, 45, 50, 60). During Fe deficiency, Fur redirects central metabolism away from Fe-requiring processes, such as respiration and the TCA cycle, and increases glycolytic and fermentative flux (60). Increasing fermentation leads to the overproduction of acidic end products, decreasing the local pH (60). *Ex vivo* studies demonstrated that reduced local pH stimulates the release of Fe ions from mammalian transferrin, suggesting that this metabolic strategy may increase Fe availability in the human host (60). The *S. aureus* respiratory complexes and the TCA cycle enzymes are two primary Fe sinks. Thus, in addition to increasing Fe availability, a shift towards fermentation enables *S. aureus* to balance its redox state and meet its energetic requirements through a metabolism less reliant on Fe.

A *S. aureus fur* mutant is sensitive to neutrophil clearance and has attenuated virulence in mouse models of pneumonia and subcutaneous skin infection (17, 45, 61). The contribution of Fur to pathogenesis has been attributed to altered expression of genes encoding virulence factors and a deficiency in the response to oxidative stress (17, 45). During infection, immune cells defend against invading bacteria by utilizing NADPH oxidase, which generates superoxide that dismutates into hydrogen peroxide, an electronically neutral molecule that can cross the cell membrane (62–64). Once cytosolic, the hydrogen peroxide can damage intracellular macromolecules, destroy solvent-exposed Fe-S clusters and mononuclear Fe sites, and catalyze Fenton chemistry using labile Fe as a catalyst (65–67). To protect against oxidative stress-induced damage, *S. aureus* encodes detoxifying enzymes, such as the heme-requiring catalase (68, 69). Fur is required for full induction of the oxidative stress response, and a *fur* mutant has reduced catalase activity, which could make cells more vulnerable to host oxidative stress clearance strategies (45, 70).

Inactivation of *fur* leads to altered expression of virulence factors, including the upregulation of cytotoxic factors (LukED, Hla, HlgC, Plc) and the repression of immunomodulatory proteins (FLIP, Spa, Efb, and superantigen-like toxins) (17). Since immunomodulatory proteins impair the recruitment of phagocytes and granulocytes, their decreased expression upon *fur* inactivation could explain why a *fur* strain is susceptible to neutrophil clearance (17). The reciprocal expression of virulence factors appears to be indirect and involves the global virulence transcriptional regulators Sae and Agr (17, 45, 70). The operons encoding for Agr and Sae lack putative Fur boxes, suggesting that their regulation by Fur is also indirect. The addition of Fe represses *sae* expression through an unknown mechanism (70). Regardless of the mechanism, these findings underscore the close relationship between Fe homeostasis and the regulation of *S. aureus* virulence factors.

## DISCOVERY OF THE IsrR AND ITS MOLECULAR ATTRIBUTES

Fur-mediated positive regulation of Fe-using processes, such as Fe storage and the TCA cycle, has been observed across bacteria and was initially revealed by the discovery of the Fur-regulated sRNA RyhB in *E. coli* (71). Under Fe-replete conditions, Fur represses *ryhB* transcription; however, during Fe limitation, Fur repression of *ryhB* is alleviated, and RyhB binds to and controls the expression of a plethora of mRNA targets by repressing translation, mediating mRNA degradation, or enhancing translation (72–74). Most RyhB mRNA targets code for Fe-using proteins such as those involved in Fe storage (FtnA, Bfr), the TCA cycle (CitB, SdhC), and reactive oxygen species detoxification (SodB) (71). Fur-regulated RyhB homologs and functional analogs have been characterized in other bacteria, and interestingly, they share similar target sets (75). Reducing the expression of Fe-requiring proteins decreases the Fe requirement under Fe limitation; therefore, RyhB regulation allows for Fe to be “spared” for essential processes. Hence, the action of RyhB and other Fur-regulated Fe-responsive sRNAs has been termed “the Fe-sparing response.”

Iron limitation or *fur* inactivation in *S. aureus* results in decreased metabolic flux through the TCA cycle and decreased catalase activity (45, 76). Both processes are shared targets of Fe-sparing sRNAs in other organisms since catalase and several TCA cycle enzymes require Fe. In *S. aureus*, the TCA cycle enzyme aconitase (AcnA) requires an Fe-S cluster to convert citrate to isocitrate, and this metabolic activity is reduced during Fe restriction (61). The finding that an *S. aureus fur* mutant has phenotypes similar to those mediated by Fur-regulated Fe-sparing sRNAs in other bacteria raised speculation for a similar type of sRNA response in *S. aureus* (75)*. S. aureus* lacks an sRNA with homology to *ryhB* or functionally similar sRNAs.

The first suggestion of a functional sRNA analog of RyhB in *S. aureus* was made in a transcriptomic study that integrated predictions of transcription factor binding sites for transcriptional regulators, such as Fur (77). The sRNA coding sequence labeled as S596 (later renamed to *isrR*) contained a potential Fur binding site, and *in silico* RNA-RNA target predictions found an enrichment of mRNAs coding for Fe-S cluster-containing proteins (77). The enrichment of Fe-associated processes in the predicted targetome led to the hypothesis that S596 was an analog of *E. coli* RyhB and the elusive mediator of the staphylococcal Fe-sparing response.

Hypothesizing the presence of a staphylococcal Fe-sparing sRNA, two groups used genetic screens to identify the sRNA IsrR in *S. aureus*. Coronel-Tellez et al. performed a fitness assay with a library of barcoded sRNA deletion mutants cultured under Fe-limited conditions (78). DNA sequencing identified a single barcoded mutant that showed a fitness disadvantage after culturing in the presence of a metal chelator. After demonstrating that the sRNA was regulated by Fur, and that it mediated the translational repression of mRNAs coding for Fe-S cluster-requiring proteins, the corresponding sRNA was renamed IsrR for “iron-sparing response regulator” (78).

A separate study leveraged the low AcnA activity phenotype of a *fur* mutant, which results in an inability to grow in chemically defined media with amino acids as the sole carbon source (61). A suppressor screen selected for strains with a second-site mutation that allowed for the growth of a *fur* mutant on amino acid medium. Genome sequencing revealed that all strains carried a mutation in the *isrR* operator. The mutations were in a DNA sequence that matched a predicted Fur-box, but they altered it from consensus (called isrR*). The isrR* alleles are unique in that the same mutation was isolated 15 times, which somehow decreases transcriptional activity of the *isrR* operator; likely through decreased recruitment of sigma factor A. The isrR* allele increased the activity of AcnA in the *fur* mutant, indicating a role for the *isrR* in repressing *acnA* expression. Deleting *isrR* in the *fur* mutant phenocopied the fur isrR* strain. To our knowledge, this is the first case of a suppressor screen identifying an sRNA by exploiting a mutant’s inability to grow under otherwise non-permissive conditions.

A global transcriptomic analysis of sRNA expression identified IsrR (also called Tsr25) as the most upregulated sRNA when cultured in human serum, suggesting a potential role for IsrR in promoting survival in blood (79). The increased *isrR* expression in human serum could be due to the presence of Fe-binding molecules, which lower *S. aureus* access to Fe and thereby alleviate Fur repression (15).

IsrR is conserved across the Staphylococcus genus (78). However, IsrR does not show homology to Fe-regulated sRNAs from other species, including the closely related Bacillales, suggesting that these sRNAs are an interesting example of convergent evolution. Similar to the results in *S. aureus*, in other staphylococci, IsrR is not expressed during growth in complex Fe-replete medium but is detected after growth in Fe-restricted media, demonstrating likely Fur regulation (78). The *isrR* gene contains two canonical Fur boxes. One in the *isrR* operator and a second overlapping the transcriptional start site. The upstream Fur box is dominant and epistatic to the second one, suggesting the presence of a second Fur box adds an extra layer of Fur repression (78).

It was recently demonstrated that sialic acid induced the expression of *isrR* independent of Fe levels (80, 81). Sialic acid control of transcription occurs via the transcriptional repressor of sialic acid catabolism, NanR. A putative NanR binding site was identified upstream of the *isrR* locus, leading to the hypothesis that sialic acid regulates *isrR* transcription. In support of this, deletion of *nanR* resulted in increased IsrR abundance. Sialic acid is catabolized to pyruvate, acetate, and fructose 6-phosphate (82). It is currently unclear why the catabolism of a carbon source that feeds into glycolysis and the TCA cycle would require the Fe-sparing response, but it could be a signal that *S. aureus* is in a specific niche. Sialic acid titers are quite high in human serum (low mM range), but it is typically covalently attached to glycoproteins or glycolipid oligosaccharides (83). Sialic acid is also found in epithelial cell membranes, suggesting a role for IsrR in colonizing the sialic acid-rich airway environment. In support of this, *isrR* was required to colonize the bronchi and lungs in a murine model of infection (61).

## IsrR TARGET PREDICTIONS AND VERIFICATION

Bioinformatic approaches are commonly used to predict putative targets of trans-acting sRNAs. A standard tool for sRNA targeting prediction is CopraRNA, which considers the evolutionary conservation of the sRNA-mRNA pairings and RNA sequence accessibility (84). To date, three studies have used CopraRNA to predict IsrR targets in *S. aureus* genomes, including one that combined CopraRNA with proteomics to predict the IsrR targetome (85). Surprisingly, the three approaches have yielded around 20 mostly distinct targets with only three targets (*acnA, fdhA, miaB*) present in all predictions (77, 78, 85). The common theme of all three IsrR targetome predictions is the enrichment of mRNAs that code for proteins that either (i) require an Fe-containing cofactor (AcnA, KatA), (ii) participate in a process involving Fe-requiring proteins (CcpE: TCA cycle, *NdhF*: respiration), or (iii) generate an Fe-containing cofactor (HemA, HemY).

Bioinformatic approaches face the challenging task of predicting sRNA-target interactions, even though these interactions often involve short, non-continuous base pairings that are influenced by intermolecular secondary structures. Therefore, sRNA target predictions from bioinformatic analysis should be complemented with direct experimental approaches. To directly identify the IsrR targetome, the Lalaouna group performed MS2 affinity purification followed by RNA sequencing (MAPS) using IsrR in *S. aureus*. MAPS consists of tagging an sRNA with the MS2 RNA aptamer, followed by sRNA-MS2 aptamer expression (86). After cytoplasmic extraction, the MS2 aptamer enables affinity purification of sRNA-target pairs, which are subsequently identified by sequencing.

IsrR MAPS analysis revealed 32 significantly enriched targets, of which one had been previously validated (*acnA* [also called *citB*]), and only three were putative targets predicted by previous bioinformatic analyses (*hemA, sodM,* SAOUHSC_00907) (87). We confirmed that IsrR bound directly to five of the MAPS targets and demonstrated IsrR control of these targets, resulting in decreased activity of Fe-associated processes in aerobic respiration, oxidative stress metabolism, and heme biosynthesis. The MAPS also enriched for targets related to purine synthesis, osmotic stress tolerance, and pathogenesis, which suggests an expanded role for IsrR beyond the Fe-sparing response. Because the MAPS and CopraRNA analyses have differing strengths and weaknesses, it has been previously noted that their overlap in putative sRNA targets is low (88). Therefore, understanding the full IsrR targetome will require a cooperative approach that incorporates bioinformatic tools and wet-lab experimental methods for direct sRNA target identification and validation.

Thus far, 22 direct targets have been validated or have experimental evidence demonstrating IsrR control (Table 1). Notably, many of the validated targets were not identified by bioinformatic analyses. Most of the validated targets are Fe-requiring gene products or participate in an Fe-related process, which aligns with IsrR function as the staphylococcal Fe-sparing mediator. Some Fe-dependent processes that appear not to be part of the IsrR regulon and might be prioritized under Fe limitation include lipoic acid synthesis (LipA), DNA synthesis and repair (MutY, AddAB, NrdG), and Fe-S cluster biogenesis (SufCDSUB).

**TABLE 1:** Verified *S. aureus* mRNA targets of IsrR

Process	Target	Reference
TCA cycle	*acnA* (*citB*)	(61, 89)
	*sdhCAB*	(61)
	*mqo*	(61)
	*citZ*	(61)
	*citM*	(61)
Aerobic respiration	*cydA*	(87)
	*cydB*	(87)
Nitrate respiration/nitrate assimilation	*narG*	(78)
	*nasD* (*nirB*)	(78)
	*gltB2*	(78)
Redox balance	*fdhA*	(78)
tRNA modification	*miaB*	(90)
ROS metabolism	*katA*	(87)
	*sodM*	(87)
Heme biosynthesis, modification, cytochrome maturation	*hemA*	(87)
	*hemE*	(87)
	*ctaA*	(87)
	*ctaB*	(87)
	*ctaM*	(87)
Mn homeostasis	*mnrS*	(91)
Arginine catabolism, urea cycle	*rocF*	(78)
Transcriptional regulation	*ccpE*	(89)

Most IsrR targets encode proteins involved in the TCA cycle and aerobic or anaerobic respiration, which rely heavily on the Fe-containing heme and Fe-S cluster prosthetic groups. Aside from the validated IsrR targets, Ganske et al. found that the TCA cycle enzyme SucAB exhibited IsrR-like regulation and the *sucAB* transcript had a predicted IsrR interaction site, suggesting that the *sucAB* mRNA is a likely IsrR target (85). The repression of TCA cycle and respiration genes by IsrR explains the Fur-dependent metabolic shift during Fe-deplete growth away from respiration and towards fermentative metabolism (16, 60, 61, 87). Whether the increase in fermentation for energy conservation is a direct or indirect result of IsrR regulation, or if it is promoted by Fur independently of IsrR, remains to be determined.

## MECHANISMS OF IsrR-DEPENDENT REGULATION

*S. aureus* IsrR is a 174-nucleotide-long trans-acting sRNA with a secondary structure consisting of three stem loops. It contains three conserved cytosine-rich regions (CRRs), each located within unpaired single-stranded regions within its secondary structure (78). CRRs are a common signature of bacterial sRNAs and are often associated with binding and occluding a target’s Shine-Dalgarno sequence, thereby preventing the formation of the ribosomal initiation complex (92). Predictions of IsrR-mRNA target interactions and targeted mutation of IsrR CRRs indicate that at least one CRR, and often two, are involved in the sRNA-mRNA pairings. The CRRs of IsrR vary in their contributions: CRR2 participates in the most interactions, and CRR3 is the most dispensable of the three (61, 78, 89, 90).

IsrR negatively impacts the expression of all its validated mRNA targets. Predictions of IsrR-mRNA interaction sites by SHAPE probing or intaRNA indicate that IsrR binding often overlaps with either the Shine-Dalgarno sequence or the mRNA translational start site (78). IsrR expression does not affect the stability of the mRNA targets *fdhA, gltB2, acnA, sdh, or miaB*, and it is associated with decreased translation or protein abundance, supporting translational control by ribosomal binding site occlusion (78, 85). In contrast to most IsrR targets, the *katA* and *rocF* transcripts demonstrate an IsrR-dependent decrease in stability during the stationary phase (85). This type of regulation by sRNAs has been shown by RyhB, which can recruit RNase E to degrade the RyhB-mRNA duplex (73). Alternatively, ribosomal occlusion by IsrR could destabilize the *katA* and *rocF* transcripts.

A recent preprint suggests that IsrR may also regulate gene expression by targeting an RNA riboswitch (91). The expression of the MntY efflux pump is regulated by the Mn-sensing MnrS riboswitch located in the *mntY* transcript 5′ untranslated region (UTR). IsrR binds to MnrS and alters its conformation. The altered conformation triggers premature transcriptional termination, leading to decreased synthesis of the *mnrSY* transcript (91). Decreased expression of MntY would presumably reduce Mn(II) export, thereby increasing intracellular Mn(II) levels. Some enzymes, such as SodM, are cambialistic and can utilize Mn(II) as a cofactor in place of Fe(II) under Fe(II)-depleted growth conditions (93). The interaction between IsrR and MnrS is an example of how IsrR employs multiple regulatory mechanisms that span both transcription and translation of its targets.

In some cases, *trans*-acting sRNAs can also promote the expression of mRNA targets. Although no IsrR-validated target is positively regulated, the combinatorial proteomics and *in silico* target-prediction approach by Ganske et al. identified two putative mRNA targets proposed to be upregulated (85). One putative target, SAUSA300_0783, is predicted to encode a histidine phosphatase, with some homology to phosphoglycerate mutase. IsrR-mediated upregulation of a phosphoglycerate mutase could contribute to the increased Fur-dependent glycolysis and fermentation rates observed during Fe-restricted growth (16, 60).

The second gene predicted to be upregulated by IsrR is SAUSA300_0324, the first gene in an operon containing *gcvH-L, lplA2,* and *sirTM (85*). Northern blot analysis after growth under Fe-deplete conditions revealed an IsrR-dependent increase in SAUSA300_0324 transcript abundance, and all operon-encoded proteins exhibited increased abundance, supporting the notion that IsrR binding to the multicistronic transcript positively influences expression (85). The genes in the *gcvH-L* operon constitute a redox-sensitive molecular switch that salvages reduced lipoic acid and promotes resistance to leukocyte-mediated oxidative stress (94). The positive IsrR-mediated expression of this operon may help promote fitness under oxidative stress.

## IsrR IMPACT ON FE IMPORT, VIRULENCE FACTOR EXPRESSION, AND PATHOGENESIS

An *isrR* mutant was attenuated for virulence in murine septicemia and acute pneumonia models, demonstrating a role for IsrR during infection across different host niches (61, 78). In a murine model of skin and soft tissue infection, an *isrR* mutant exhibited no significant decrease in lesion size or general necrosis, but it did demonstrate decreased colonization (61). The requirement for IsrR in pathogenesis during septicemia and pneumonia, but not in skin infection, may result from dioxygen availability in these tissues. In aerobic environments, Fe(II) is oxidized to insoluble Fe(III), limiting its availability. Conversely, in microaerophilic environments, the rate of Fe(II) oxidation is lower, increasing bioavailability and decreasing the demand for the Fe-sparing response. IsrR could contribute to pathogenesis by promoting Fe acquisition or controlling the expression of virulence factors, as suggested by the MAPS and proteomics data (60, 87, 95).

Promoting Fe uptake is a common function of Fe-responsive sRNAs. For example, RyhB increases the expression of mRNAs (*shiA, cirA*) involved in Fe acquisition (74, 96). Whole-cell metal quantification in *S. aureus* demonstrated that *isrR* expression during *fur* inactivation is associated with increased cell-associated Fe and increased siderophore production (61). Proteomic analyses determined that *isrR*-deficient strains had lower abundances of select Isd proteins (IsdB, IsdC, IsdD, IsdE, IsdG), staphylopine (CntA), and the staphyloferrin B transporter (SirA) (95). *In silico* IsrR targetome predictions have not identified mRNAs encoding Fe uptake systems, suggesting that IsrR support of Fe acquisition may be indirect.

One mechanism by which IsrR could indirectly impact Fe uptake is by decreasing *ccpE* expression (89). CcpE regulates heme and siderophore Fe uptake; therefore, IsrR repression of *ccpE* or the depletion of citrate, the CcpE co-activator, could lead to increased expression of Fe uptake genes (97, 98). An alternate hypothesis is that IsrR mediates Fe uptake by repressing heme biosynthesis. Heme accumulation suppresses the synthesis of staphyloferrin B through a mechanism involving heme transfer from IsdG to SbnI, which subsequently binds to DNA and regulates the *sbn* staphyloferrin B biosynthetic locus (99). Heme-bound SbnI represses *sbn,* while apo-SbnI activates its expression (100). It remains to be determined whether and how endogenous heme impacts SbnI enzymatic activity.

Analysis of the secretome from IsrR-expressing cells revealed that IsrR activates the expression of several Sae-regulated virulence factors (95). During the exponential growth phase, IsrR expression was associated with increased expression of immunomodulatory factors (Ecb, Efb, Sbi, Ssl), while in the stationary phase, IsrR was associated with increased expression of cytotoxic factors (Nuc, SplB, HlgC, Hla). IsrR expression was positively associated with increased alpha hemolysin (Hla) and enhanced hemolytic activity (95). The activation of Sae is thought to be indirect, as IsrR is not predicted to interact with the *saePQSR* transcript. IsrR also promoted the expression of Sae-independent virulence factors (SdrC, SdrD, ScpA) (95). IsrR exemplifies the crucial interplay between central metabolism and virulence, and future work will elucidate the mechanism underlying IsrR control of virulence factor expression.

## ACONITASE HAS A BIFUNCTIONAL ROLE AS AN RNA-BINDING PROTEIN (RBP) DURING Fe LIMITATION

In both eukaryotes and bacteria, conditions that decrease the occupancy of the AcnA Fe-S cluster, such as during Fe restriction, increase the abundance of apo-AcnA, which can function as an RBP. The first described bifunctional AcnA was the eukaryotic cytosolic iron regulatory protein 1 (IRP1) (101, 102). IRP1 switches between the holoenzyme with metabolic activity during Fe-replete growth and the apoenzyme with RBP function when Fe is scarce (103). IRP1 controls mRNA stability and translation by binding to iron regulatory elements (IREs) located in the 5′ or 3′ UTRs of target mRNAs (104). Eukaryotic genes containing IREs are involved in the uptake, transport, or utilization of Fe, demonstrating that AcnA can be a moonlighting enzyme with a regulatory role in Fe homeostasis (104).

Eukaryotic and bacterial AcnA share conserved amino acids essential for their activities, and a bifunctional AcnA has been described in various bacteria, including *E. coli* (105), *B. subtilis* (106), and *S. aureus* (89). *E. coli* can synthesize two aconitases (AcnA, AcnB), and both have RBP function in their apo-form. AcnA and AcnB regulate the expression of their own transcripts, as well as the *sodA* mRNA (105, 107). In *B. subtilis*, the AcnA RBP decreases the stability of the citrate synthase (*citZ*) transcript by binding to its 5′ end (106). Reduced CitZ synthesis prevents citrate accumulation, which can chelate cytosolic Fe(II) (106). Although eukaryotic IRE stem loops contain the consensus sequence CAGUG, no consensus sequence has been determined for prokaryotic apo-AcnA RBP binding (108).

During Fe-deplete growth, *S. aureus* AcnA functions as an RBP (89). Barrault et al. demonstrated that the AcnA RBP and metabolic activities function independently. The AcnA RBP negatively impacted mRNA levels of *citZ, citC,* and its own transcript (89). Additionally, RBP activity increased the abundance of the *pycA* transcript, which encodes pyruvate carboxylase. PycA catalyzes an anaplerotic reaction that replenishes oxaloacetate (OAA), a substrate required to initiate the TCA cycle and a precursor for the synthesis of various metabolites. Citrate can also bind to holo-AcnA and protect the Fe-S cluster from oxidative or nitrosative damage (109–111).

The regulatory effects of IsrR and apo-AcnA RBP illustrate how *S. aureus* goes to great lengths to suppress the TCA cycle during Fe depletion. In the case of *acnA* expression, the transcript is repressed directly by IsrR, indirectly by IsrR translational control of the transcriptional activator CcpE, and by the apo-AcnA RBP, which binds its own transcript to decrease expression (89). Aside from decreasing non-essential sources of Fe usage, TCA cycle repression by IsrR could paradoxically decrease citrate, which is a precursor for the synthesis of both staphyloferrins. Although staphyloferrin B can be synthesized from citrate derived from the Fe-regulated citrate synthase SbnG (encoded within the *sbn* biosynthetic locus), staphyloferrin A is dependent on CitZ-derived citrate (112). Intracellular citrate accumulation can chelate Fe(II); therefore, decreasing citrate titers during Fe scarcity could be a way to prevent unnecessary cytosolic Fe sequestration (97). Decreasing citrate would also impact CcpE activity, which uses citrate as a co-activator for transcriptional activation (98). CcpE is predicted to control the expression of over 100 genes, including those involved in central carbon processing, Fe uptake, and virulence (97); therefore, modulating CcpE transcriptional activation by controlling citrate pools may promote *S. aureus* survival during Fe depletion.

## CONCLUSION

**Fig 2:**
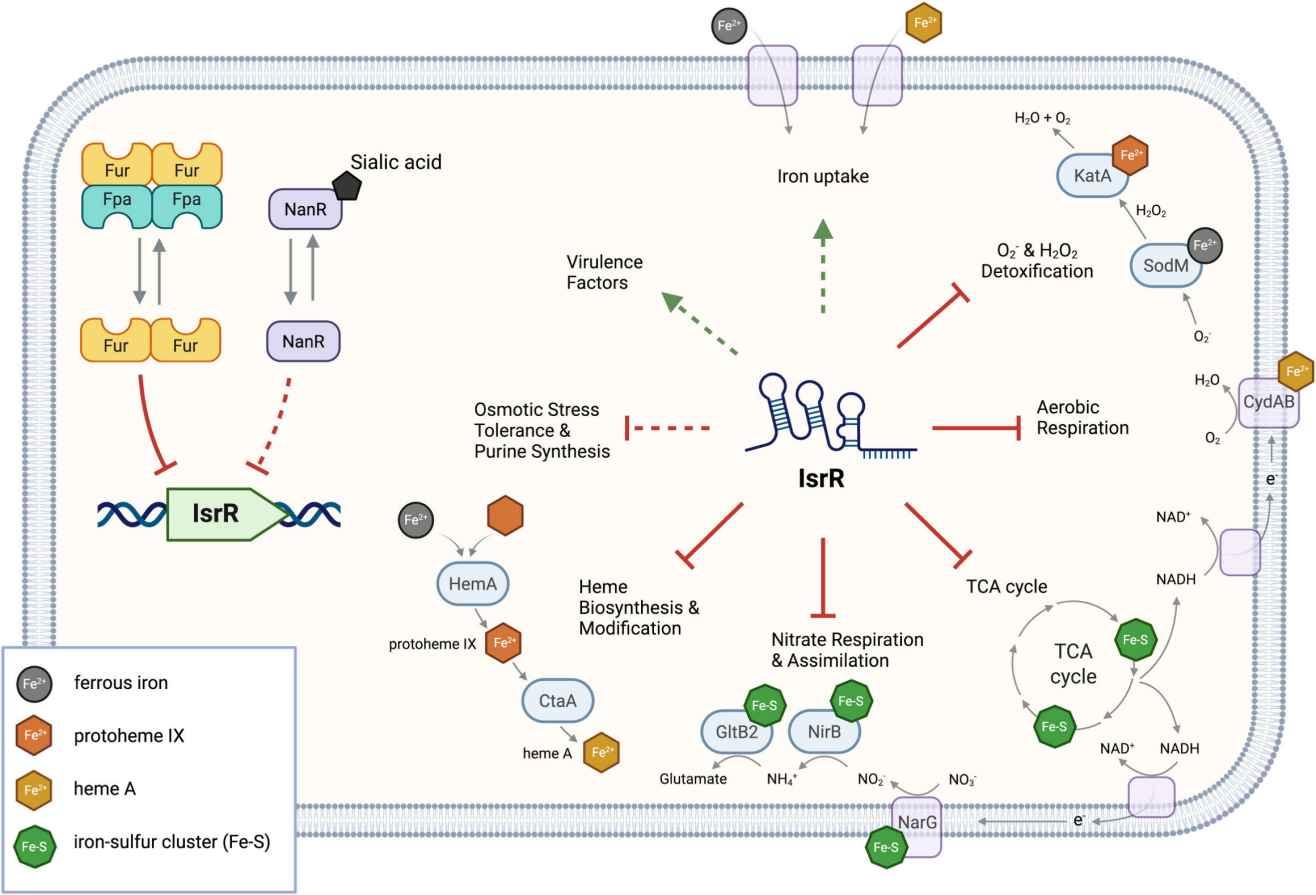
Model for IsrR transcriptional regulation and physiological impacts. IsrR is repressed under Fe-replete conditions by Fur and is also predicted to be controlled by NanR in the absence of sialic acid. During Fe-limiting conditions, Fur repression is alleviated using Fpa, and IsrR is expressed. IsrR mediates an Fe-sparing response that represses translation of mRNAs encoding Fe-utilizing proteins. IsrR represses the expression of mRNAs encoding proteins involved in the detoxification of superoxide (O_2_^−^) and hydrogen peroxide (H_2_O_2_), the TCA cycle, aerobic respiration, nitrate respiration and assimilation, heme biosynthesis, modification, and cytochrome maturation. IsrR is also predicted to bind and repress mRNAs involved in osmotic stress tolerance and purine synthesis. Experimental evidence suggests that IsrR promotes Fe acquisition and the expression of cytotoxic virulence factors; however, the mechanisms are unknown. The green and red lines indicate positive and negative regulation, respectively, while the dashed lines represent an unclear regulatory mechanism. Created in BioRender (G. Rios, 2026. https://BioRender.com/oid7tz1).

The studies and findings summarized herein highlight the significant roles of *fpa*, *acnA*, *fur*, and *isrR* in regulating *S. aureus* physiology to adapt to Fe-deplete conditions. Upon Fe scarcity, the concerted action of these regulatory gene products results in increased expression of Fe uptake mechanisms, decreased Fe-reliant respiratory metabolism, increased glycolytic and fermentative metabolism, reduced oxidative stress metabolism, and altered expression of virulence factors (Fig. 2). The importance of these regulators is underscored by findings that, across different infection models, Fur and IsrR contribute to *S. aureus* pathogenesis. Continued investigation into the Fur-Fpa regulatory interplay, the IsrR and apo-AcnA RBP targetomes, and the mechanism by which Fur and IsrR impact virulence factor expression is necessary to fully understand the adaptive strategies that *S. aureus* utilizes when faced with host-mediated Fe limitation.

The vast impact of IsrR on *S. aureus* physiology emphasizes the importance of sRNAs as key regulators of stress responses. The utility of sRNAs is highlighted by their ability to counteract transcription factor regulation and impact preexisting messages without requiring protein synthesis. Understanding the regulatory mechanisms of bacterial transcription factors and sRNAs will improve our understanding of bacterial physiology under stressful conditions, such as those encountered in host niches.
